# Genomes of three tomato pathogens within the *Ralstonia solanacearum *species complex reveal significant evolutionary divergence

**DOI:** 10.1186/1471-2164-11-379

**Published:** 2010-06-15

**Authors:** Benoît Remenant, Bénédicte Coupat-Goutaland, Alice Guidot, Gilles Cellier, Emmanuel Wicker, Caitilyn Allen, Mark Fegan, Olivier Pruvost, Mounira Elbaz, Alexandra Calteau, Gregory Salvignol, Damien Mornico, Sophie Mangenot, Valérie Barbe, Claudine Médigue, Philippe Prior

**Affiliations:** 1 CIRAD, UMR Peuplements Végétaux et Bioagresseurs en Milieu Tropical, Saint Pierre F-97410, La Réunion, France; 2 Université de Lyon, Unité Mixte de Recherche, Centre National de la Recherche Scientifique, UMR CNRS 5557 Ecologie Microbienne, IFR41 Bio Environnement et Santé, Université Lyon I, Villeurbanne cedex F-69622, France; 3 CNRS-INRA, Laboratoire Interactions Plantes-Microorganismes, UMR2594, BP52627, Castanet-Tolosan F-31326, France; 4 AgroParisTech, ENGREF, 19 avenue du Maine, Paris F-75732, France; 5 University of Wisconsin-Madison, Department of Plant Pathology, 1630 Linden Drive, Madison, WI 53706, USA; 6 Biosciences Research Division, Department of Primary Industries, 475 Mickleham Rd, Attwood, VIC, 3049, Australia; 7 CNRS-UMR 8030, Laboratoire d'Analyse Bioinformatique en Génomique et Métabolisme, Commissariat à l'Energie Atomique (CEA), Direction des Sciences du Vivant, Institut de Génomique, Genoscope, 2 rue Gaston Crémieux, 91057 Evry Cedex, Evry cedex F-91006, France; 8 Commissariat à l'Energie Atomique (CEA), Direction des Sciences du Vivant, Institut de Génomique, Genoscope, 2 rue Gaston Crémieux, 91057 Evry Cedex, Evry cedex F-91006, France; 9 INRA-CIRAD, UMR Peuplements Végétaux et Bioagresseurs en Milieu Tropical, Saint Pierre F-97410, La Réunion, France

## Abstract

**Background:**

The *Ralstonia solanacearum *species complex includes thousands of strains pathogenic to an unusually wide range of plant species. These globally dispersed and heterogeneous strains cause bacterial wilt diseases, which have major socio-economic impacts. Pathogenicity is an ancestral trait in *R. solanacearum *and strains with high genetic variation can be subdivided into four phylotypes, correlating to isolates from Asia (phylotype I), the Americas (phylotype IIA and IIB), Africa (phylotype III) and Indonesia (phylotype IV). Comparison of genome sequences strains representative of this phylogenetic diversity can help determine which traits allow this bacterium to be such a pathogen of so many different plant species and how the bacteria survive in many different habitats.

**Results:**

The genomes of three tomato bacterial wilt pathogens, CFBP2957 (phy. IIA), CMR15 (phy. III) and PSI07 (phy. IV) were sequenced and manually annotated. These genomes were compared with those of three previously sequenced *R. solanacearum *strains: GMI1000 (tomato, phy. I), IPO1609 (potato, phy. IIB), and Molk2 (banana, phy. IIB). The major genomic features (size, G+C content, number of genes) were conserved across all of the six sequenced strains. Despite relatively high genetic distances (calculated from average nucleotide identity) and many genomic rearrangements, more than 60% of the genes of the megaplasmid and 70% of those on the chromosome are syntenic. The three new genomic sequences revealed the presence of several previously unknown traits, probably acquired by horizontal transfers, within the genomes of *R. solanacearum*, including a type IV secretion system, a rhi-type anti-mitotic toxin and two small plasmids. Genes involved in virulence appear to be evolving at a faster rate than the genome as a whole.

**Conclusions:**

Comparative analysis of genome sequences and gene content confirmed the differentiation of *R. solanacearum *species complex strains into four phylotypes. Genetic distances between strains, in conjunction with CGH analysis of a larger set of strains, revealed differences great enough to consider reclassification of the *R. solanacearum *species complex into three species. The data are still too fragmentary to link genomic classification and phenotypes, but these new genome sequences identify a pan-genome more representative of the diversity in the *R. solanancearum *species complex.

## Background

The rapidly accumulating complete genomes in databases provide unique opportunities to study relationships among organisms. Since DNA sequences are conserved between closely related organisms, comparative genomic analyses are a powerful tool for understanding the complex evolutionary events in specific phylogenetic lineages.

*R. solanacearum*, formerly known as *Pseudomonas solanacearum *and *Burkholderia solanacearum*, is the causal agent of bacterial wilt [1]. This soil-borne vascular pathogen is widely distributed in tropical and subtropical climates and affects an unusually broad range of crops, including both monocot and dicot plants [2-4]. Many affected hosts are critical for developing countries because of their strategic importance as cash crops or as subsistence foods like potato (*Solanum tuberosum*), tomato (*S. lycopersicum*), eggplant (*S. melongena*), cooking banana (*Musa spp.*) and peanut (*Arachis hypogea*). In the 1990s, potato brown rot strains of *R. solanacearum *historically known as race 3/biovar 2 (r3b2) were introduced in Europe and North America [5,6]. Due to their adaptation to tropical highland climates, these strains, which are more virulent at cool temperatures (~20°C) than tropical strains [7], may pose major threats in temperate zones. Therefore, *R. solanacearum *was listed as a quarantine organism in Europe and Canada and as a Bioterrorism Select Agent in the U.S. [8].

*R. solanacearum *and the closely related species *R. syzygii *(a pathogen of clove in Indonesia) and the banana blood disease bacterium (BDB) form a complex in the *R. picketii *lineage [9-12]. This species complex includes thousands of genetically distinct strains that can differ from each other by more than 30%, and thus do not belong in the same species by conventional definition [13]. This species complex includes strains with broad and narrow host ranges, which are ecologically different as well: potato strains are cold-tolerant and banana strains are insect-transmitted, and with different geographic origins. Because *R. solanacearum *strains have been isolated from virgin jungle soils in both Asia and the Americas, the origin of the species complex is believed to predate the geological separation of the continents [14]. Based on analyses of the 16S-23S internal transcribed spacer (ITS) region, *egl *and *hrpB *genes and on comparative genomic hybridization (CGH), the *R. solanacearum *species complex is hierarchically classified in four phylogenetic groups called phylotypes, which reflect their origins as follows: Asia (phylotype I), the Americas (phylotype II), Africa (phylotype III) or Indonesia (phylotype IV, which includes *R. syzygii *and BDB) [15,16]. Each phylotype can be further subdivided into sequevars, or sequence variants, which may contain isolates with similar virulence patterns or common geographic origin [12].

Despite their considerable phylogenetic diversity, *R. solanacearum *strains are unified by their common pathology. All cause bacterial wilt disease, which is characterized by bacterial colonization of the plant xylem vessels to very high cell densities (10^9 ^- 10^10 ^CFU/ml xylem fluid), vascular browning, stunting, wilting, and often rapid death [3]. The bacterium is transmitted by soil, surface water, and infected propagation materials like potato tubers or ornamental cuttings. It most commonly infects plants through the roots, but some strains are insect-transmitted [17]. Bacterial wilt is difficult to control because the bacterium survives for years in infested soils and weed hosts. Breeding for host resistance, the best management strategy is complicated by the pathogen's high genetic diversity. For example, tomatoes resistant to *R. solanacearum *strains in one region are often susceptible to those in another [18].

Genome sequences of *R. solanacearum *strains can answer historic questions about what traits allow this bacterium to be such an aggressive and lethal pathogen of so many different plants, and to survive in such different habitats as soil, water, non-host plant rhizospheres and host xylem vessels. The bi-partite genome of *R. solanacearum *strain GMI1000 (phylotype I, sequevar 18) was sequenced and analyzed [19,20] . The genome has two replicons, called the chromosome and the megaplasmid, with a mosaic structure that implies many rearrangements and horizontal gene transfers. Several factors have been shown to contribute to bacterial wilt virulence, especially the type III secretion system (TTSS) and associated effectors [21], and production of extracellular polysaccharides and enzymes [22]. The GMI1000 genome encodes more than a hundred TTSS effectors or putative effectors [23]. Draft genomes are available for two additional *R. solanacearum *strains: IPO1609/UW551, which cause potato brown rot disease in cool-temperate climates and Molk2, which causes Moko disease of banana and plantain [24,25]. These respectively belong to phylotype IIB sequevar 1 (IIB-1) and sequevar 3 (IIB-3).

To better understand how this highly diverse and scattered species complex has evolved and diverged without losing its fundamental pathological qualities, we sequenced the genomes of three additional broad host range strains from other phylotypes, namely: American strain CFBP2957 (IIA-36), African strain CMR15 (III-29) and Indonesian strain PSI07 (IV-10). All were originally isolated from tomato. Their complete genomes were manually annotated and analyzed with the aim of investigating the conserved, variable, and specific gene repertoires of these strains and the three previously sequenced ones, with a special emphasis on genes involved in virulence and pathogenicity. In addition, the genomes of 51 *R. solanacearum *strains (including the six sequenced strains) were compared by CGH on a pan-genomic microarray. These comparative genomic approaches produced new insights into the evolution and taxonomy of the *R. solanacearum *species complex.

## Results and Discussion

It has long been known that *R. solanacearum *is a highly heterogeneous group of strains and thus no one genome sequence could represent the entire species complex. Initial genomic studies revealed that *R. solanacearum *strains have a substantial backbone of common housekeeping and virulence functions, but also carry a divergent set of genomic modules that likely confer distinct ecological phenotypes and host specificity [19,24]. Comparing multiple bacterial genomes distributed around the phylogenetic tree was therefore essential to understand the evolutionary driving forces and mechanisms that have produced such a phenotypically and genotypically diverse group. Four existing genome sequences represented phylotype I (tomato isolate GMI1000) and phylotype II (IPO1609/UW551 and Molk2, from potato and banana, respectively) [19,24,25]. The phylotype-sequevar subclassification system provided rational criteria for choosing more strains to sequence to cover significant additional diversity in the species complex. We therefore sequenced genomes of strains that cause bacterial wilt of tomato from Indonesia (PSI07, phylotype IV), Cameroon (CMR15, phylotype III), and the French West Indies (CFPPB2957, phylotype II).

### Overview of genomes of CFBP2957, CMR15 and PSI07

The two-replicon (chromosome and megaplasmid) genome architecture of these three tomato strains was identical to that found in the three previously sequenced strains. As in the GMI1000 genome [20], most -but not all- housekeeping genes were carried on the chromosome. However, the capture or creation of the megaplasmid by *Ralstonia *spp. appears to be an ancient event, since all strains in the *R. pickettii *lineage studied to date have two replicons (Lucas et al, unpublished). Moreover, CGH microarray analyses by Guidot et al. [16] established that both replicons have a long history of coevolution within the *R. solanacearum *species complex.

The sizes of the chromosomes and megaplasmids, and therefore the entire genome, are similar in all strains The average genome size for the sequenced *R. solanacearum *strains was approximately 5.7 Mb including the chromosome (3.6 Mb) and the megaplasmid (2.1 Mb) (Table 1). Additional data on genome characteristics are provided in supplemental material [Additional file 1]. *R. solanacearum *strains CFBP2957, CMR15 and PSI07 had genome lengths of 5,683,402 bp, 5,606,288 bp and 5,593,041 bp, respectively, with a common average G+C content of 66.7% in both replicons. With an average protein coding density of 86.8%, chromosomes contained about 3500 predicted coding sequences (CDS), and megaplasmids contained about 1800. Only one rRNA operon was detected in each *R. solanacearum *genome, except in strains GMI1000 and CMR15, which had 4 and 3 rRNA operons, respectively. Additional ribosomal operons can permit faster adaptation to new environmental conditions by increasing protein synthesis capacity [26]. In CMR15, two rRNA operons were on the chromosome and one on the megaplasmid; GMI1000 has three on the chromosome and one on the megaplasmid. The three CMR15 rRNA operons were 99.5% identical at the nucleotide level. Fifty-nine, 49 and 56 tRNA genes were identified in strains CFBP2957, CMR15 and PSI07, respectively.

**Table 1:** General features of genomes of *R. solanacearum *strains CFBP2957, CMR15, PSI07, GMI1000, IPO1609 and Molk2

	Phyl.	Geo. Ori.	length	GC%	#CDS	rRNA op	tRNA	Ref. strain	Ref. genome.
CFBP2957	IIA	French West Indies	5,683,402	66.9%	5310	1	56	[55]	This study
CMR15	III	Cameroon	5,593,041	66.9%	5149	3	59	[56]	This study
PSI07	IV	Indonesia	5,606,288	66.3%	5247	1	49	[12]	This study
GMI100	I	French Guyane	5,810,922	67.0%	5635	4	57	[76]	[19]
IPO1609	IIB	Nederland	5,523,292	66.7%	5203	1	Na	[77]	[24]
Molk2	IIB	Indonesia	5,862,101	66.7%	5438	1	Na	[24]	[24]

		Mean	5,679,841	66.7%	5330	1.7	47.3		

Data for chromosomes and megaplasmids are combined. For more details, see Table S1. (Phyl. = phylotype, Geo. Ori = geographical origin, #CDS = number of coding sequences, rRNA op = number of rRNA operons, tRNA = number of tRNA, Na = Not available.)

Automatic re-annotation of the *R. solanacearum *sequences in the public domain identified 517 additional coding sequences (CDS) in GMI1000 (263 on the chromosome and 254 on the megaplasmid [Additional file 2]). These newly identified genes, encoding mostly proteins of unknown function, were encoded 'RALSO' to distinguish them from previously-annotated CDS ('RSc' and 'RSp').

### The *R. solanacearum *pan-genome

**Figure 1:**
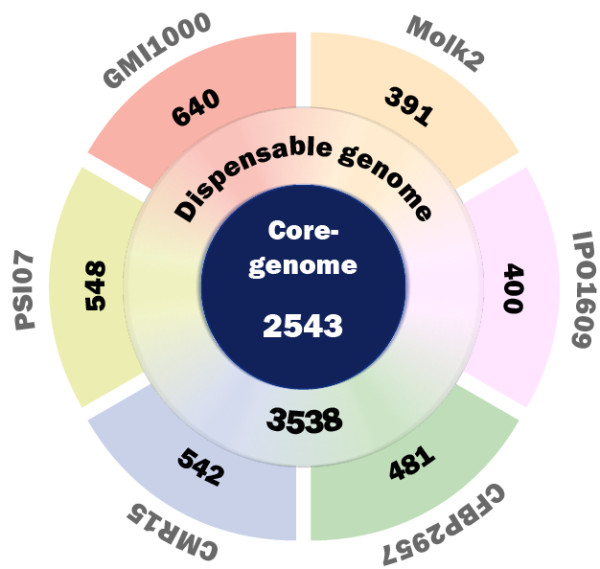
**Number of genes in the *R. solanacearum *pan-genome**.

The new genome sequences from strains CMR15, CFBP2957 and PSI07 were combined with existing sequences from strains GMI1000, IPO1609, and Molk2 to identify a total of 9093 unigenes which constitute the known pan-genome of *R. solanacearum*: that is, the set of all genes present in a group of organisms. The pan-genome is composed of the core-genome (genes present in all strains), the dispensable genome (genes present in some strains, but not all) and the specific genome (unique genes present in only one strain) [27]. As reported in figure 1, among these 9093 genes, 2543 were highly conserved (bidirectional best hit (BBH)) in the six genomes and constituted the *R. solanacearum *core-genome, which thus makes up 28% of the pan-genome. Ninety-three percent of the core genome CDS were located on the chromosome. The dispensable genome contained 3538 genes (39% of the pan-genome). This is in general agreement with a previous conserved genome estimate of 2690 genes that was based on CGH analyses of 18 strains [16]. The overestimate by the CGH approach is probably due to nonspecific hybridizations and lower technical precision of hybridization compared to sequencing. The number of strain-specific genes was variable, ranging from 391 for Molk2 to 640 for GMI1000 (figure 1). For these six *R. solanacearum *strains, a total of 3012 genes were identified as strain-specific genes, which represents a third of the current pan-genome. Depending on the strain, the number of proteins of unknown function (or conserved proteins of unknown function) encoded by the strain-specific genes ranged from 73 to 84% in. These genes likely hold many clues to the traits and mechanisms underlying the biological diversity of the *R. solanacearum *species complex, but because so few of these CDS can be functionally annotated, gene by gene mutagenesis and phenotype analysis will be needed to determine their roles.

### Genome plasticity and genomic islands

**Figure 2:**
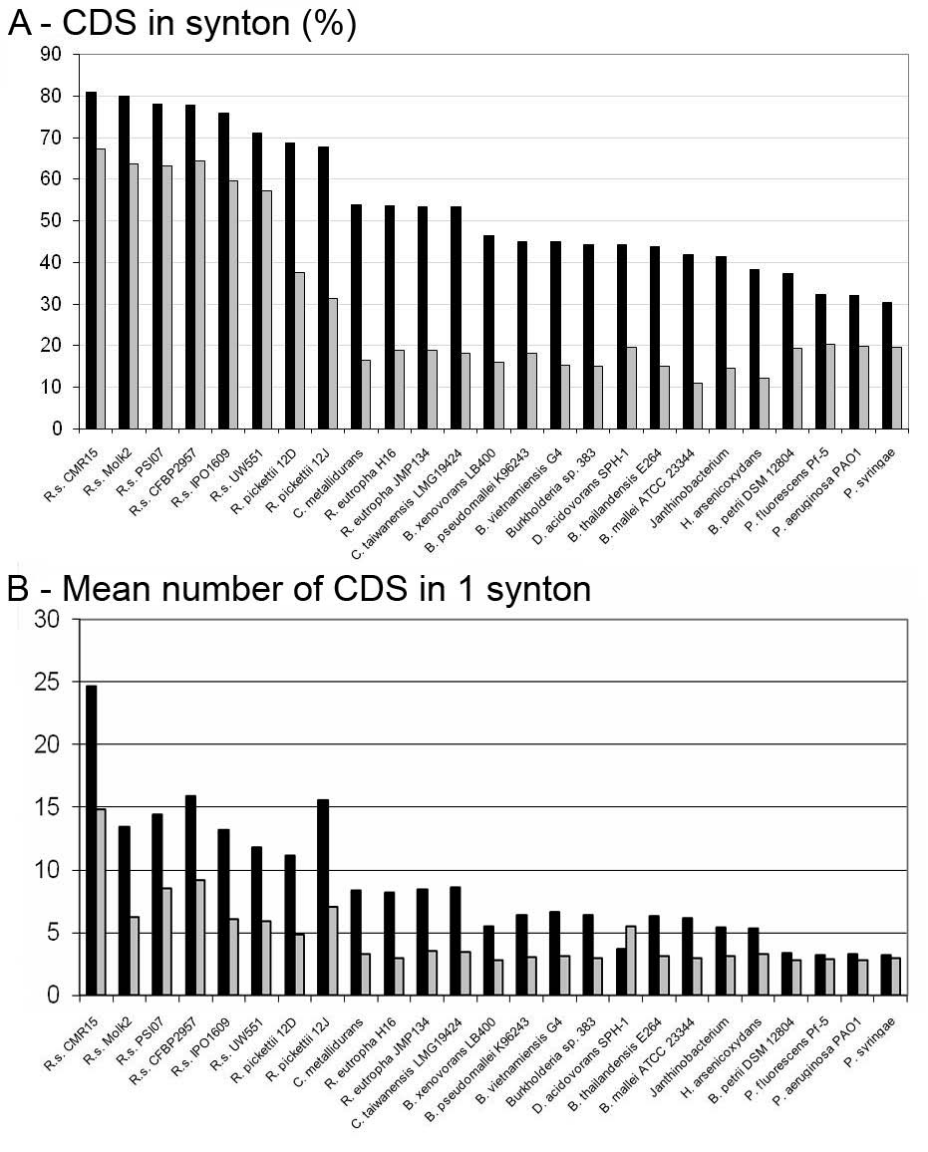
**Synteny in the *R. solanacearum *species complex**. Figure 2A shows the percentage of CDS in synteny in each strain compared with strain GMI1000. Figure 2B shows the mean number of CDS in 1 synton compared with GMI1000. Results for chromosome and megaplasmids are depicted in black and grey, respectively.

**Figure 3:**
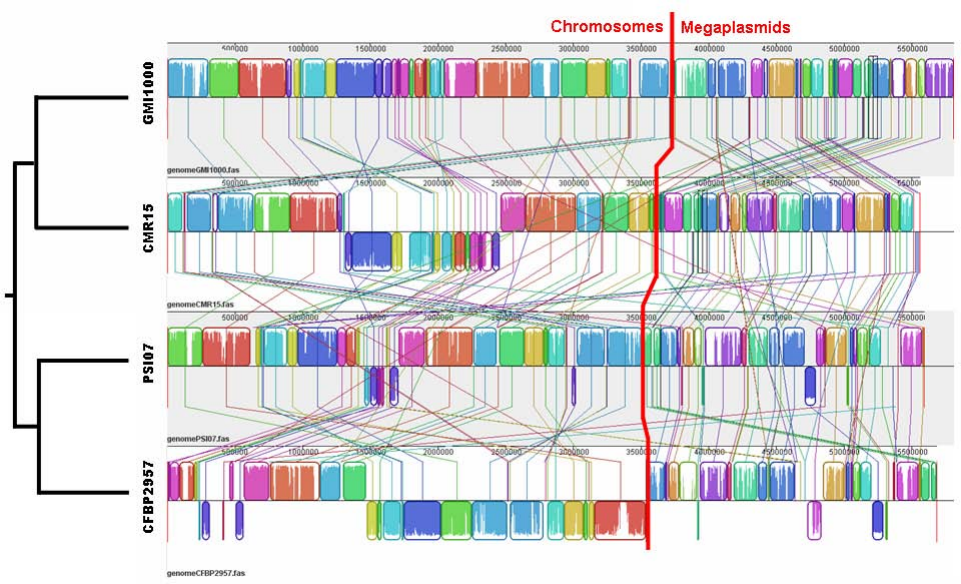
**Multiple genome alignment for strains GMI1000, CMR15, PSI07 and CFBP2957**. Fine colored lines represent rearrangements or inversions relative to the GMI1000 genome. Chromosomes are separated from megaplasmids by the thick red line.

Working from the GMI1000 genome, we identified syntenic CDS in all six *R. solanacearum *genomes and also in some bacterial species most closely related to the *R. picketii *lineage (figure 2A). The six *R. solanacearum *genomes were highly syntenic: 55 to 65% of the CDS on the megaplasmid and 70 to 80% of the CDS on the chromosome were in synteny. This percentage decreased to below 55% on the chromosome and 20% on the megaplasmid in other closely related species, except for *R. picketii*, where 68 and 40% of CDS, on the chromosome and the megaplasmid respectively, were in synteny with GMI1000. *R. pickettii *is closely related to, but not a member of the *R. solanacearum *species complex; both belong to the *R. pickettii *lineage (as distinct from the *R. eutropha *lineage) [9]. *Cupriavidus *spp. and *R. eutropha *are the closest relatives to the *R. pickettii *lineage, but in these genomes the number of CDS in synteny with GMI1000 was dramatically lower on both replicons. Further, the size of syntenic regions was greater within the *R. solanacearum *species complex; an average of 11 to 16 CDS per synton was observed on chromosomes of these strains (figure 2B, black bar). The exception was the African strain, CMR15, where we found an average of 25 CDS per synton with GMI1000. For the other related species, the mean number of CDS in one synton was fewer than 8. For all comparisons, we found that the megaplasmids always contained fewer CDS in synteny than the chromosomes. Multiple alignments of *R. solanacearum *genomes revealed that many genomic rearrangements occurred in the history of these organisms, including intra- and inter-replicon rearrangements (figure 3).

**Figure 4:**
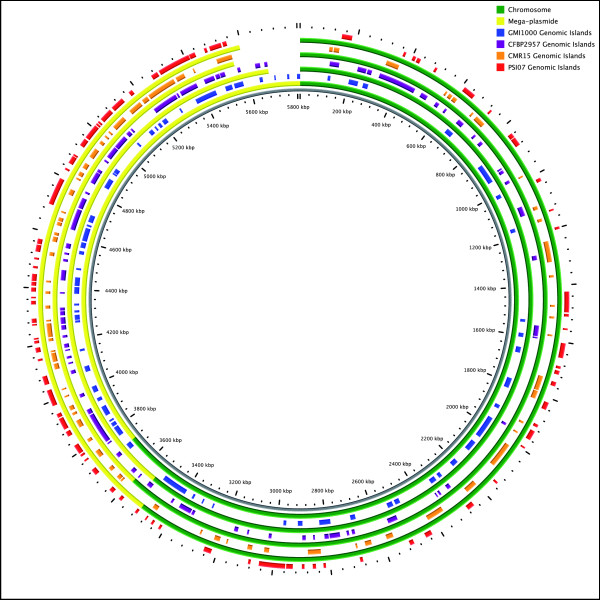
**Localization of the principal Genomic Islands detected in strains GMI1000, CMR15, CFBP2957 and PSI07**. From inside to outside: genomic islands from GMI1000 (in blue), from CFBP2957 (in purple), from CMR15 (in orange) and from PSI07 (in red). Chromosomes and megaplasmids are represented in green and yellow, respectively.

*R. solanacearum *is naturally competent for DNA transformation [28-30] which allows cells to acquire foreign genetic material directly from their environment. Nakamura et al [31] estimated that nearly 16% of GMI1000 genes were horizontally transferred. Genomic islands are parts of genomes that display evidence of horizontal acquisition. They have a minimal length of 5 kb and contain CDSs with no BBH and no synteny with genomes of compared organisms. Table S3 [Additional file 3] provides a complete list of the numerous genomic islands detected in CFBP2957, CMR15 and PSI07, and a schematic representation of genomic island locations is given in figure 4. The density of genomic islands was two-fold greater on megaplasmids than on chromosome. Many of these genomic islands were IS or phage sequences containing almost exclusively proteins of unknown function. However, some genomic islands carried type III effectors (or putative effectors), including, in CMR15: *GALA8*, an unknown effector, similar to other GALA effectors (GR1 on the chromosome); *popP2 *(GR11 on the chromosome); as well as putative effectors (GR29 or GR32 on the megaplasmid). Two supplementary rRNA operons (GR12-chromosme and GR2-megaplasmid) and *nosZRDFYL*, an operon involved in the anaerobic denitrification pathway (GR12-megaplasmid) were also located on genomic islands in CMR15. In PSI07, three putative type III effectors were detected on chromosomal genomic islands (GR12 and GR31) and one on the megaplasmid (GR38). In CFBP2957, only two putative effectors were detected in genomic islands (GR4-chromosome and GR3-megaplasmid). These results are still too fragmentary to determine any relationship between host ranges and the presence or absence of repertoires of specific effectors. However, acquisition of new effectors could theoretically contribute to rapid adaptability and diversification, especially in virulence and aggressiveness, as well as to saprophytic fitness.

Interestingly, the megaplasmids of CFBP2957 and PSI07 also harbor a genomic island containing the *rhi *operon. This operon was composed of 9 genes: *rhiB *(20274 pb), *rhiC *(97185 pb), *rhiI *(867 pb), *rhiD *(12468 pb), *rhiH *(1458 pb), *rhiE *(12657 pb), *rhiF *(7824 pb), *rhiA *(7158 pb) and *rhiG *(1983 pb). The *rhi *genes were previously unknown in *Ralstonia *strains, but are present in *Pseudomonas fluorescens *[32] and in *Burkholderia rhizoxinica *where they encode a non-ribosomal peptide synthase (NRPS) that synthesizes the antimitotic toxin rhizoxin [33]. Although this operon spans more than 80 kb, it could have been horizontally acquired since *R. solanacearum *can exchange and integrate DNA fragments of 30 to 90 kb by natural transformation [29]. The insertion of such a toxin operon in the genome of *R. solanacearum *could provide a competitive advantage in the soil environment.

### Comparison of some metabolic properties

**Figure 5:**
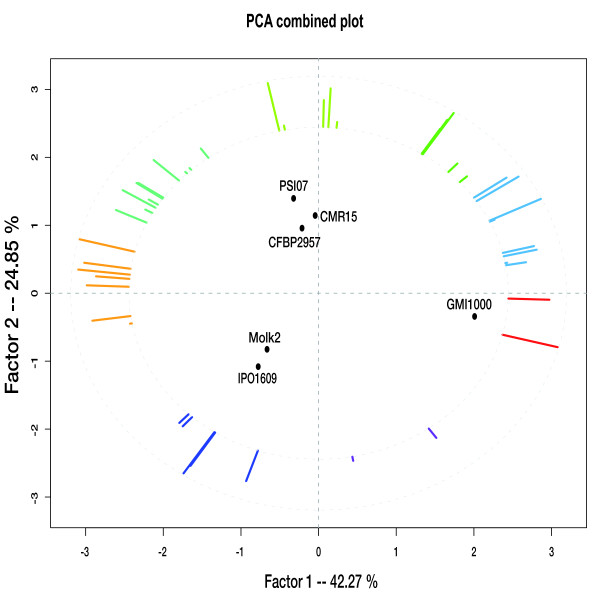
**Principal Component Analysis of six *R. solanacearum *genomes, performed from a two dimensional matrix, combining genomes and metabolic pathways**. Individual points represent genomes, and colored vectors symbolize completion of some pathways (number of reaction for pathway x in a given organism/total number of reactions in the same pathway x defined in the MetaCyc database) in the data (see method section). Pathways with similar completions (vectors with similar orientation) have been clustered and drawn in a same color. Thus, genomes can be associated with their representative and characteristic groups of metabolic pathways (i.e. vectors pointing in their direction). The corresponding pathway functions are listed in Suppl.Table S4.A.

To better understand metabolic diversity among the sequenced strains, we used Principal Compenents Analysis (PCA), a type of factorial analysis that analyzes a data matrix by several quantitative variables [34]. We applied PCA to a matrix describing the completion (measured as a percentage) of all known metabolic pathways present in the six *R. solanacearum *genomes (figure 5). The first two resulting factors captured over 67% of the data's total variability. The axes were interpreted with the help of external information, such as each strain's host, phylotype, and the laboratory of annotation. The first factorial axis separated strain GMI1000 from the five other strains. This result accounted for the metabolic functions corresponding to the red-colored vectors. The list of pathways that these vectors represent can be consulted in Table S4 A and B [additional file 4], and generally have a maximum of two or three reactions. None of these pathways appear to be complete in GMI1000. This suggests that this factorial axis is in fact an artifact of insufficient or erroneous enzymatic function annotations. This should help guide future annotation efforts. The second factorial axis separated the strains according to their hosts: banana and potato for Molk2 and IPO1609, and tomato for PSI07, CMR15 and CFBP2957. As would be expected, Molk2 and IPO1609, which are close together in the factorial plane, actually belong to the same phylotype (IIB).

Careful examination of the metabolic potential of the studied genomes has revealed some specificity capabilities for compound degradation. Although *R. solanacearum *is a highly adapted and ancient plant pathogen, this organism can also survive well in soil and rhizospheres and, consequently, its genome encodes ability to metabolize diverse organic compounds as energy sources. Pathway analyses indicate that this bacterium can utilize a wide range of substrates including amino acids, sugars, and fatty acids as well as glycerol, ethanol, methylglyoxal, and beta-ketoadipate ([Additional file 4, sheet B], also Genin and Boucher [20]). GMI1000 was predicted to grow on various aromatic compounds including ferulate, vanillate, and protocatechuate [20], which are released when lignin degrades in soil. However strains CMR15 and GMI1000 do not harbour the *benABCD *and *catABC *genes involved in benzoate and catechol degradation to beta-ketoadipate. Another contrasting example is D-galactonate degradation, which can serve as energy and sole carbon source for many enteric bacteria [35]. In soil, bacteria such as *Azotobacter vinelandii *and *Sinorhizobium meliloti *convert D-galactose to D-galactonate via the De Ley-Doudoroff pathway, and finally to D-glyceraldehyde-3-phosphate and pyruvate. Our data indicate that D-galactonate degradation is functional only in strains GMI1000, CMR15 and PSI07 [Additional file 4, sheet B]. However, this substrate must be taken up whole from the environment because no degradation enzymes upstream in the pathway were apparent in these genomes. Curiously, it seems that the D-galactonate transport system is different in CMR15, which harbors only one likely transport gene in the corresponding synton, and in GMI1000 and PSI07, which each have 3 genes highly similar to the L-arabinose ABC transporter (annotated as *araFGH*).

Urease, which is necessary for utilization of urea as sole nitrogen source, has three main subunits and five accessory proteins. Genetic determinants for this enzymatic activity were found in all strains except Molk2, the only banana wilt pathogen sequenced to date. All six strains appear able to metabolize inorganic nutrients such as sulfate and nitrate, consistent with experimental data [14]. However, the denitrification pathway was complete only in strains GMI1000 and CMR15, because the *nosZ *gene encoding nitrous oxide reductase was absent from the four other strains; this heterogeneity was previously noted [25]. Genes for periplasmic nitrate reductase (*nap*), nitrate reductase (*nar*), nitric oxide reductase (*nor*) and nitrite reductase (*nir) *were present on the megaplasmids of all six sequenced *R. solanacearum *strains.

Finally, all six genomes contain genes involved in the detoxification of noxious compounds and in metal resistance, which likely support colonization and survival in specific ecological niches. An interesting example is arsenic resistance which in bacteria is mediated in part by *ars *genes. Among these, *arsC *encodes an arsenate reductase [As(V)→As(III)], *arsA *and *arsB *encode an arsenite efflux pump, and *arsR *encodes a transcriptional regulator [36]. As(III) is known to induce oxidative stress, to cause DNA damage, and to inhibit the DNA repair system [37]. It is generally further oxidized by the arsenite oxidase [As(III)→As(V)] encoded by *aoxAB *genes. The *arsC *gene is the sole gene in this pathway present in all six strains, with two tandem copies in PSI07. In addition, only PSI07 can oxidize arsenite: on the PSI07 megaplasmid is a cluster containing two *arsC *genes, *aoxAB*, and *arsR*. The annotation of the *arsC-like *gene is probably erroneous in the other *Ralstonia *species (and in many other bacterial genomes as well).

### Virulence factors

Many traits contribute to virulence of *R. solanacearum *strains. The best known are the type III secreted effectors, well described in this bacterium and in other plant pathogens [23]. However, other traits, such as production of EPS and cell wall-degrading enzymes, are also important for wilt disease development. Based on the literature, we created an inventory of 128 genes involved in virulence from the six sequenced *R. solanacearum *genomes [Additional file 5: Supplemental Table S5]. Some genes are involved in swimming motility, twitching motility and chemotaxis. Table S5 gives a representative pair of genes for each of those functions. Virulence genes were subdivided into 5 categories: type III effectors (TTE) and putative effectors, the exopolysaccharide (EPS) biosynthetic genes, the cell wall-degrading enzyme (CWDE) genes, response to host defence genes and key virulence regulators. Scrutiny of the genomes shows that all six strains have all genes needed for functional type II and III secretion systems. Similarity distances between each sequenced strain were computed on the basis of gene presence/absence data for these 128 virulence genes (data not shown). Phylogenetic analysis constructed on the basis of: 1) all known or putative Type 3 effector genes in the pan-genome, and 2) all known virulence factor genes of all kinds in the pan-genome, resulted in trees that were significantly different from each other, and significantly different from trees based on well-conserved genes like *mutS *and *egl *sequences, or on the entire genome sequences (data not shown). This result suggests that virulence factor genes have evolved or been lost or added at substantially different rates than *R. solanacearum *genes as a whole. A more fine-scale case-by-case analysis will likely be needed to trace the evolutionary history of individual virulence traits. Analysis of strains hosted by plants phylogenetically distant from tomato (a common host for all strains analyzed here except Molk2) may elucidate roles of individual virulence factors in determining host range.

Type III-secreted effectors are an important potential source of host range variability in *R. solanacearum *strains; these have mainly been described in GMI1000 and UW551 to date [23,25]. Specific effectors that are important in CMR15, CFBP2957 and PSI07 are unknown. We attempted to detect new type III effectors with the Effective software [38], but this did not work well for *R*. *solanacearum *strains, giving about 50% false negative on previously annotated effectors (data not shown).

### Plasmids in *Ralstonia solanacearum *strains

Genin and Boucher [20] suggested that the presence of small plasmids in *R. solanacearum *cells, initially described by Morales and Sequeira [39], was more an exception than a rule. However, we found small plasmids (<50 kb) in the African and Indonesian strains. These plasmids were named pRSC35 (35 kb, GC% = 61.3) in strain CMR15 and pRSI13 (12.8 kb, GC% = 61.0) in strain PSI07. The presence of small plasmids is therefore maybe less rare in *R. solanacearum *strains than previously thought. These small plasmids may have remained undetected until now because their very low copy number makes them difficult to purify (unpublished results).

Despite their low copy numbers, the stability of these plasmids is apparently ensured by two different toxin/antitoxin (TA) systems. On pRSC35, two CDS had a limited homology with zeta-toxin and epsilon-antitoxin (<40%), which form a post-segregational mechanism for plasmid maintenance in bacteria [40,41]. The regulator ω was not detected in the CMR15 genome. This zeta/epsilon TA system is well described and a similar system confers a bactericidal effect on *Bacillus subtilis*, and bacteriostatic effects on *E. coli *[42].

**Figure 6:**
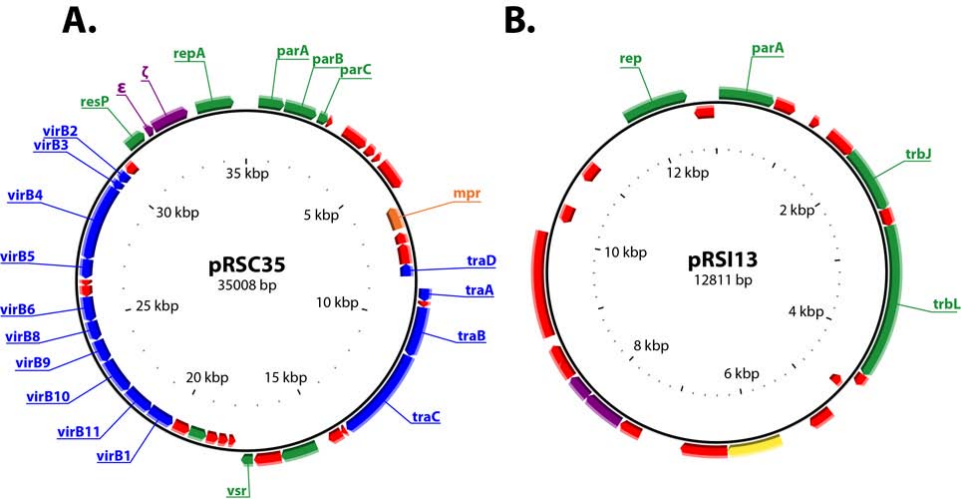
**Circular representation of plasmids pRSC35 and pRSI13**. A: pRSC35, B: pRSI13; in blue: Type IV Secretion System; in green: proteins involved in DNA metabolism; in purple: toxin/antitoxin systems; in red: proteins of unknown function; in orange: the metalloprotease gene of pRSC35.

The plasmid pRSC35 was broadly syntenic with plasmids from many plant-associated bacteria including pXcB of *Xanthomonas citri *pv. *aurantifolii *(65% of CDS in synteny), diverse *P. putida *plasmids (from 58 to 62% synteny), a *X. citri *pv. *citri *plasmid (58% synteny) and a plasmid from *X. euvesicatoria *(51% synteny). Among the 44 CDS present on this plasmid (figure 6A), 14 appeared to be involved in the Type IV secretion system: 10 genes make up the *virB *operon (*virB1, 2, 3, 4, 5, 6, 8, 9, 10 *and *11*) ranging from 5 to 15 kbp, and four genes form the *tra *operon (*traA, B, C *and *D*) from 28 to 34 kbp. Eight CDS coded for proteins potentially involved in DNA metabolism (such as partition proteins *parA*, *parB *and *parR*, a DNA methyltransferase, and a DNA mismatch endonuclease). Finaly, one CDS had a strong homology to a Zn-metalloprotease (*mpr*), also carried on plasmids in several human and/or animal pathogenic bacteria or opportunistic bacteria: *P. putida*, *Yersina pestis*, *Escherichia coli *O157:H7, *Klebsiella pneumoniae*, *Salmonella enterica*, *etc*. Metalloproteases like those encoded on pRSC35 are essential for the infection process of many eukaryotes [43-46].

The unexpected Type IV Secretion System is unique among *R. solanacearum *strains studied to date and could play diverse important roles in virulence and adaptation. The CMR15 Type IV secretion system genes, which are clustered together with the *virB *operon, have nearly the same organization as on pXAC64 of *Xanthomonas citri *pv *citri *[47]. The type IV secretion system is a bacterial conjugation apparatus [48] and the DNA thus efficiently imported through the cell envelope can directly increase the fitness or virulence of bacteria by mediating the acquisition of new traits like effectors or antibiotic resistance genes. Type IV secretion systems can also be directly involved in virulence via direct injection of effectors or DNA into plant cells [49,50]. No obvious type IV effectors were found on pRSC35 or in the complete genome of CMR15, but some proteins of unknown function could be Type IV effectors. Additional experiments are needed to investigate (i) the distribution of this plasmid in African phylotype III strains, (ii) the ecological and pathogenic role of this plasmid in the phenotype of phylotype III strain CMR15, and (iii) the occurrence of such plasmids in strains belonging to other phylotypes.

A second low-copy number plasmid, pRSI13, was present in PSI07. It was syntenic with a plasmid found in *Nitrobacter hamburgensis *X14 (34% of CDS in synteny), *Burkholderia pseudomallei *9 and 91 (30 and 26% respectively), *Parvibaculum lavamentivorans *DS-1 (26%), *Acidovorax *sp. JS42 (26%) and *E. coli *pOLA52 (26%). pRSI13 contained 23 CDS, 16 of which encoded for proteins of unknown function and one for a putative transcriptional regulator. Other pRSI13 CDS coded for proteins putatively involved in DNA metabolism or conjugation (figure 6B). Thus, the functional annotation reveals no obvious role for this plasmid in either the ecology of the bacteria (saprophytic life in the soil) or during pathogenesis. The maintenance of this plasmid seems likely due to the TA system rather than to increased fitness.

### New insight into the phylogeny of the *R. solanacearum *species complex

**Figure 7:**
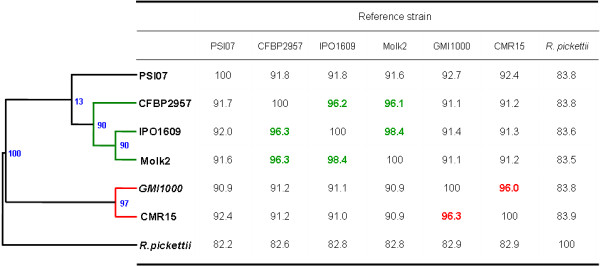
**Phylogeny based on pairwise comparison of average nucleotide identity (ANI)**. Strains grouped with an ANI value >95% are in the same color. Bootstrap values are indicated in blue. ANI analyses were conducted using perl scripts (Konstantinidis and Tiedje, 2004).

Genomes were compared pairwise using the average nucleotide identity (ANI) calculation (figure 7); the dendrogram summarizes the results, which grouped together strains GMI1000 (Asia) and CMR15 (Africa), with ANI values above 96%. New World (American) strains CFBP2957, Molk2 and IPO1609 (phylotype II) were likewise clustered together (above 98% ANI between Molk2 and IPO1609 and above 96% between CFBP2957 and Molk2-IPO1609). Indonesian strain PSI07 was closest to the phylotype II group, but the ANI values between PSI07 and any other strain were always less than 95%. The topology of the species complex phyogenetic tree computed from ANI values was fully consistent with that observed in previous trees computed with CGH microarray data [16] and with *mut*S and *hrp*B sequences [15]. Thus, working with a smaller number of strains but with much more data per strain, these genome sequences confirm the phylotype classification scheme and their phylogenetic position, i.e. phylotype I is closest to phylotype III, and phylotype IV is closest to the phylotype IIA and IIB. Kanstantinidis and Tiedje [51] and Goris et al. [52] demonstrated that ANI values above 95% are equivalent to the 70% DNA-DNA hybridization cut-off value traditionally used to differentiate bacterial species. Using this standard, our data identify three evolutionarily distinct groups within the *R. solanacearum *species complex: GMI1000-CMR15; CFBP2957-IPO1609-Molk2; and strain PSI07. Separate ANI analysis of replicons gave the same result as whole genome analyses (data not shown). As proposed by Stakebrandt et al [53], genomic data can be used to propose new species, provided that there is sufficient congruence with DNA-DNA reassociation.

**Figure 8:**
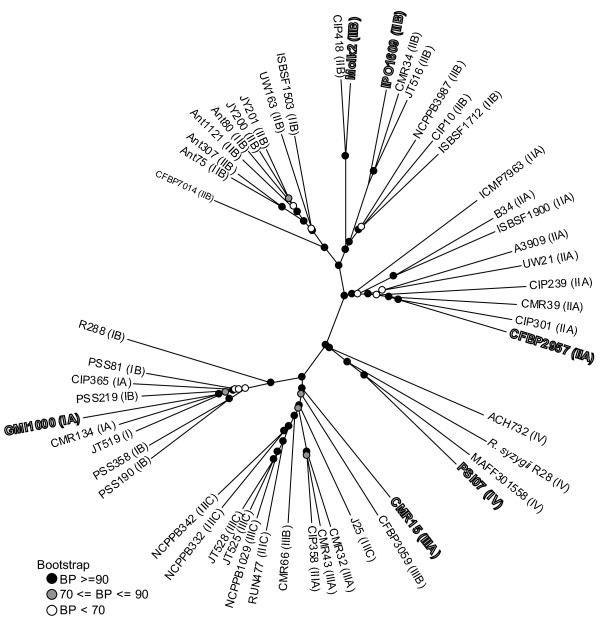
**Hierarchical clustering of 51 *R. solanacearum *strains from CGH results**. The six sequenced strains are lined by a thin black label. Bootstrap values are figured by grayscale circles.

We used CGH of a spotted microarray reflecting the pan-genome of GMI1000, IPO1609, and Molk2 to further define phylogenetic relationships among a set of 51 strains selected to span the known diversity within the species complex, and to verify that the three new genomes selected were truly representative of their respective phylotypes. Hierarchical clustering of these 51 strains (figure 8) differentiated five clusters, which each cluster matching to a phylotype (or a phylotype subdivision). This dendrogram is fully congruent with the validated phylogeny of the species complex [15,54] and deepens a previous analysis performed on a smaller set of strains with a less complete CGH microarray based only on the GMI1000 genome [16]. Furthermore, the position of each sequenced strain within its own phylotype cluster allowed us to extend ANI results to the other 46 strains. The taxonomy of the species complex can now be reviewed based on these consistent results from several different techniques. The analyse of genetic distance between genome sequences of much strain, especially in phylotype IV, should be decisive, but our CGH data suggest that there is a solid phylogenetic basis for dividing this group into three species, according to the phylotype scheme: one species containing Phylotype II, a second containing Phylotypes I and III, and a third containing *R. solanacearum *strains from Phylotype IV.

## Conclusion

We compared the genome organizations and gene repertoires from six phylogenetically distant and phenotypically divergent strains from the *R. solanacearum *species complex. These strains shared many structural and genotypic traits observed in the first sequenced strain of *R. solanacearum*, GMI1000. Our results significantly expanded the known *R. solanacearum *pan-genome, identifying thousands of genes that more sharply define the common core, the dispensable and the specific (strain-unique) genomes. However, the genomes differed quite significantly. Our ANI and other analyses suggest that this large and heterogeneous species complex spans enough variation that it could be reclassified into at least three distinct taxonomic groups, which each have the equivalent of more than 30% divergence from the other two at the DNA-DNA hybridization level. Post-genomic mining of this pan-genome can combine comparative tools like CGH microarrays with phenotypic experiments to determine the distribution and the biological functions of likely traits identified with *in silico *analyses. Such combined approaches will increase our understanding of the evolutionary past, the phylogeography, and the biological specialization of *R. solanacearum *species complex strains. Although four of the six strains sequenced to date were isolated from tomato plants, our analysis did not identify any variations in previously known virulence factors that were unique to tomato pathogens. This could be explained by either 1) an insufficiently large sample of non-tomato pathogen genomes or 2) a biological unity in the core mechanisms of bacterial wilt across all *R. solanacearum *species complex members, with host specificity and ecological adaptations conferred by traits that remain to be identified. Sequencing of additional species complex members that infect highly divergent plant hosts such as clove trees and plantains will offer additional insights into the traits that confer host specificity on bacterial wilt pathogens.

## Methods

### Strains

The three sequenced strains were isolated from infected tomato plants (*Solanum lycopersicum*) in different geographic locations: CFBP2957 (phylotype IIA-36) was isolated in the French West Indies [Syn. MT5, [55]], CMR15 (phylotype III-29) in Cameroon [56] and PSI07 (phylotype IV-10) in Indonesia [12]. Bacteria were grown at 28°C in B liquid medium [57]. Strains CFBP2957, PSI07 (CFBP7288) and CMR15 (CFBP6941) were deposited at CFBP [58] (*Collection Française de Bactéries Phytopathogènes*, Angers, France). Table S6 [Additional file 6] provides a list of the 51 *R. solanacearum *strains used in microarray experiments, with their geographical origin and host of origin.

### Sequencing and assembly

Genomic DNA was purified from overnight liquid cultures of each strain using a DNeasy Blood & Tissue Kit (Qiagen, Hilden, Germany), according to the manufacturer's recommendations. Sequencing of the *R. solanacearum *strains CMR15, CFBP2957 and PSI07 was performed using the strategy described by Aury et al. [59]. Around 20× coverage of 454 GSflx reads were mixed with 1× coverage Sanger reads for the scaffolding, which was derived from a 10 kb insert fragment size library. Each library was constructed after mechanical shearing of genomic DNA and cloning of generated inserts into plasmid pCNS (pSU18-derived). Plasmid DNAs were purified and end-sequenced (11520 *R. solanacearum *PIII, 14592 *R. solanacearum *PIIA and 7680 *R. solanacearum *PI) by dye-terminator chemistry with ABI3730 sequencers (Applied Biosystems, Foster City, USA) leading to an approximately 1-fold coverage. The sequences were assembled using Newbler (Roche Diagnostics) and validated via the Consed interface [60]. For the finishing phases, we used primer walking of clones and/or PCRs and transposon bombs Template Generation System™ II Kit (TGS™ II Kit) (Finnzyme), Kan3 as well as around 60× coverage using Solexa reads GAI to polish the genome draft.

### Automatic and expert annotation of the *Ralstonia *genomes

Coding sequences (CDS) were predicted using AMIGene (Annotation of Microbial Genomes) software [61]. Each predicted CDS was assigned a unique identifier prefixed with "CMR15_", "CMR15_mp" and "pCMR15_", for *R. solanacearum *CMR15 (respectively the chromosome, megaplasmid, and plasmid), with "PSI07_", "PSI07_mp" and "pPSI07" for *R. solanacearum *PSI07 (respectively the chromosome, megaplasmid, and plasmid), and with "RCFBP_", "RCFBP_mp" for *R. solanacearum *CFBP2957 (respectively the chromosome and megaplasmid). The set of predicted genes were submitted to automatic functional annotation using the tools listed in Vallenet et al. [62]. Apart from the plasmid-encoded genes, the functional assignment was first based on the reference genome of *Cupriavidus taiwanensis *annotations [63] for strong orthologs i.e., 85% identity over at least 80% of the length of the smallest protein. All these data (syntactic and functional annotations, and results of comparative analysis, see below) are stored in a relational database, called RalstoniaScope. Manual validation of the automatic annotation was performed using the web-based MaGe [64] (Magnifying Genomes) interface, which allows graphic visualization of the annotations enhanced by a synchronized representation of synteny groups in other genomes chosen for comparison. As described by Vallenet et al. [65] the system also offers several functions to guide accurate manual expert annotation. We performed a complete manual annotation of the CMR15 genome and then used it to automatically annotate strong orthologs in PSI07 and CFBP2957. Only 'specific' regions of these two strains, *i.e*. those containing genes not orthologous to ones in CMR15, were manually annotated. Finally, this expert work was used to update the annotation of GMI1000, which was published in 2002, and to automatically annotate the two other sequenced (but not finished) strains, Molk2 and IPO1609 [24]. Using the available contigs of Molk2 and IPO1909, we were not able to properly organize the corresponding sequences using GMI1000 as a reference genome. Genomes of these two *R. solanacearum *strains are thus not correctly assembled in MaGe and some analyses remain impossible with these genomes.

Complete sequence data for CFBP2957, CMR15, PSI07, GMI1000, Molk2 and IPO1609 are publicly available via the MaGe interface (RalstoniaScope [66]). Sequences and annotations data of *R. solanacearum *CFBP2957, CMR15 and PSI07 have also been deposited at the EMBL database [67] [EMBL:FP885897 and EMBL:FP885907 (chromosome and megaplasmid), EMBL:FP885895 and EMBL:FP885896, and EMBL:FP885906 and EMBL:FP885891 respectively. Sequences of plasmids pRSC35 and pRSI13 are available using accession numbers EMBL:FP885893 and EMBL:FP885890].

### Genomic Island Identification

We used the *RGPfinder *tool in the MaGe annotation platform (Roche et al., in preparation) to investigate Regions of Genomic Plasticity (RGPs) in the whole genome sequences of *R. solanacearum *GMI1000, CMR15, PSIO7, CFBP2957, Molk2 and IPO1609. RGPs are defined as regions of at least 5 kb that are missing in at least one of the genomes compared. This definition makes no assumption about the evolutionary origin or genetic basis of these variable chromosomal segments. *RGPfinder *searches for synteny breaks between a target genome and a set of closely related bacteria (generally other strains) to define RGPs. It also provides information about composition abnormalities (%G+C deviation, Codon Adaptation Index) and RGP flanking features such as tRNA, IS and repeats, which are common characteristics of genomic islands (GI). Moreover the tool integrates the results of Alien Hunter [68] a method that analyses compositional biases to detect atypical sequences (*i.e*., sequences potentially acquired by horizontal gene transfer).

### Synteny group computation

Sequence data for comparative analyses were obtained from the NCBI database (RefSeq section [69]). Putative orthology relationships between two genomes were defined by gene pairs satisfying either the BBH criterion or an alignment threshold (at least 40% sequence identity over at least 80% of the length of the smallest protein). These relationships were subsequently used to search for synteny groups (*i.e.*, conservation of the chromosomal co-localisation between pairs of orthologous genes from different genomes) among several bacterial genomes using an algorithm based on an exact graph-theoretical approach [70]. This method allowed for multiple correspondences between genes, detection of paralogy relationships, gene fusions, and chromosomal rearrangements (inversion, insertion/deletion). The 'gap' parameter, representing the maximum number of consecutive genes that are not involved in a synteny group, was set to five.

### Average nucleotide identity calculation

The average nucleotide identity (ANI) was calculated according to Konstantinidis and Tiedje [51]. Pairwise comparisons between sequences were done separately for chromosomes and megaplasmids (GMI1000, CFBP2957, CMR15 and PSI07), and then for entire genomes (GMI1000, Molk2, IPO1609, CFBP2957, CMR15 and PSI07). Similarity between strains (using Euclidian distance) and dendrogram computation were conducted with the R statistical environment [71], using ape and ade4 libraries [72,73].

### Metabolic network comparison

The metabolic network of each genome was predicted by the "Pathway Tools" software [74] using MetaCyc [75] as a reference pathway database (version 12.0). Starting with the functional annotation performed in MaGe, this software applies selection rules to infer possible metabolic pathways and builds a special database called a PGDB (Pathway/Genome Database). These metabolic networks for each *R. solanacearum *genome are directly available in the MaGe graphical interface.From those data, a two-dimensional matrix was built, wherein each line represents a *Ralstonia *genome, and each column a specific pathway measure (according to the Metacyc metabolic classification). Each value corresponds to a pathway completion measure (defined as the number of enzymatic reactions which have been found in a given pathway divided by the total number of reactions in this pathway in Metacyc). This data matrix is the starting point of Principal Component Analysis, which highlights possible metabolic similarities and specificities between the genomes.

All *R. solanacearum *genomes were considered in this statistical analysis, but only pathways with non-constant completion could be analyzed. After examination of the amount of inertia captured by the method's resulting factors, 2 axes were kept for further analysis (they represent more than 67% of the total dispersion). For graphical representations, the variable (pathways) plot and the individual (genomes) plot were combined, restricting plotted variables to those with a quality of representation greater than 0.75, in order to conserve interpretability. Solely as an additionnal aid to interpretation and to listing readability, for each factorial plane, the pathway variables were hierarchically clustered according to the angles between their vectors. The clustering method used a Euclidean distance and ward's criterion; the number of classes was chosen after manual examination of the cluster tree, and led to 8 classes for the first factorial plane (see also Table S4-A).

### CGH microarray experiments

The DNA microarray used in these experiments was generated by C. Boucher and collaborators (INRA-CNRS, Toulouse, France). This spotted microarray consists of 6,516 65-mer and 70-mer oligonucleotides representative of the genes identified from the genomes of *R. solanacearum *GMI1000 [EMBL:AL646052.1 and EMBL:AL646053.1], IPO1609 [GenBank:NW_002196569.1] and Molk2 [GenBank: NW_002196564.1]. Each gene was represented by a single oligonucleotide except for 115 effector genes, which were represented by two to six oligonucleotides to distinguish allelic forms of a given gene. A limited number of oligonucleotides representative of particular intergenic regions were also included on the microarray. This microarray also includes a set of appropriate negative controls. Each oligonucleotide was spotted twice on the microarray. DNA extraction and labelling, and microarray hybridization were performed as described by Guidot et al [16]. Standard control DNA used for all genome hybridization experiments consisted of an equimolar combination of the genomic DNA from the three sequenced strains GMI1000, IPO1609 and Molk2. Analysis was conducted as previously described using ImaGene and GeneShight (BioDiscovery) softwares [16]. A gene was considered as absent from the tested strain when the base 2 logarithm of the ratio of the normalized hybridization signal of the tested strain over the normalized hybridization signal with the control DNA was lower than the cutoff value of -1 [28].

## Abbreviations

ANI: Average Nucleotide Identity; BBH: Bidirectional Best Hit; BDB: Blood Disease Bacterium; CDS: CoDing Sequence; CFBP: Collection Française de Bactéries Phytopathogènes; CFU: Colony Forming Unit; CGH: Comparative Genomic Hybridization; CWDE: Cell Wall Degrading Enzymes; EPS: Exopolysaccharides; GI: Genomic Island; GR: Genomic Region; ITS: Internal Transcribed Spacer; NRPS: Non-Ribosomal Peptide Synthase; PGDB: Pathway/Genome Database; r3b2: race3/biovar2; RGP: Region of Genomic Plsaticity; TTE: Type Three Effectors; TTSS: Type Three Secretion System.

## Authors' contributions

BR, AG, CA, MF, VB, CM and PP wrote the paper. AC, GS, DM, SM, VB and CM performed genomes sequencing and assembly. AG and ME performed CGH microarrays. BR, BCG, AG, GC, MW and PP carried out manual annotation. BR, CA, OP, GS, DM, CM and PP analyzed data. All authors read and approved the final manuscript.

## Supplementary Material

Additional file 1 **genomes overview**. Main features of CMR15, CFBP2957, PSI07, GMI1000, Molk2 and IPO1609 genomes. Click here for file

Additional file 2 **new CDS GMI1000**. New CDS detected in GMI1000 chromosome (sheet 1) and megaplasmide (sheet2). Click here for file

Additional file 3 **Genomics Islands**. Genomic Island predicted in CMR15 (sheet 1 and 2), CFBP2957 (sheet 3 and 4) and PSI07 (sheet 5 and 6) genomes. Click here for file

Additional file 4 **Metabolic pathways**. Sheet A: class of each pathway class according to figure 6. Sheet B: Pathway completion. Click here for file

Additional file 5 **Virulence genes**. CDS involved in virulence. Click here for file

Additional file 6 **strains (microarray)**. Strains used in CGH microarray experiments. Click here for file
